# The disparities in health insurance ownership of hospital-based birth deliveries in eastern Indonesia

**DOI:** 10.1186/s12913-021-07246-x

**Published:** 2021-11-22

**Authors:** Agung Dwi Laksono, Ratna Dwi Wulandari, Zuardin Zuardin, Nopianto Nopianto

**Affiliations:** 1 National Institute of Health Research and Development, the Ministry of Health of the Republic of Indonesia, Jakarta, Indonesia; 2 Persakmi Institute, Surabaya, Indonesia; 3 Faculty of Public Health, Universitas Airlangga, Surabaya, Indonesia; 4 Faculty of Psychology and Health, UIN Sunan Ampel, Surabaya, Indonesia; 5 STIKes Tengku Maharatu Tengku Maharatu, Pekanbaru, Indonesia

**Keywords:** Health insurance, Maternity care, Maternal health, Woman health, Eastern Indonesia

## Abstract

**Background:**

Development in Eastern Indonesia tends to be left behind compared to other Indonesian regions, including development in the health sector. The study aimed at analyzing the health insurance ownership disparities in hospital delivery in Eastern Indonesia.

**Methods:**

The study draws on secondary data from the 2017 Indonesia Demographic and Health Survey. The study population was women aged 15–49 years who had given birth in the last five years in Eastern Indonesia. The study analyzes a weighted sample size of 2299 respondents. The study employed hospital-based birth delivery as a dependent variable. Apart from health insurance ownership, other variables analyzed as independent variables are province, residence type, age group, marital status, education level, employment status, parity, and wealth status. The final stage analysis used binary logistic regression.

**Results:**

The results showed that insured women were 1.426 times more likely than uninsured women to undergo hospital delivery (AOR 1.426; 95% CI 1.426–1.427). This analysis indicates that having health insurance is a protective factor for women in Eastern Indonesia for hospital delivery. There is still a disparity between insured and uninsured women in hospital-based birth deliveries in eastern Indonesia. Insured women are nearly one and a half times more likely than uninsured women to give birth in a hospital.

**Conclusion:**

The study concludes that there are health insurance ownership disparities for hospital delivery in eastern Indonesia. Insured women have a better chance than uninsured women for hospital delivery.

## Introduction

World Health Organization (WHO) notes that, in the case of births and delivery or within 42 days of pregnancy termination, mother mortality (MMR) is the annual number of female deaths due to all factors related to or caused by or for the period and site of the pregnancy. Except for causes of accident or event [1]. One of the Sustainable Development Goals (SDGs) goals is to reduce the MMR globally to less than 70 per 100,000 live births by 2030 [2].

Referring to the Ministry of Health of the Republic of Indonesia’s annual report in 2019, maternal deaths were 4221 cases. This number has decreased slightly compared to 2018, which amounted to 4226 cases. It consists of maternal mortality caused by bleeding in 1280 instances, hypertension in pregnancy in 1066 cases, infection in 207 patients, circulatory system disorders in 200 cases, metabolic disorders in 157 points, and other causes 1311 cases [3].

Every day 38 mothers in Indonesia die from diseases/complications related to pregnancy and complications [4]. We could have been preventing most of these deaths. It means that we could avoid and saved if the MMR was high. Indonesia’s MMR trend has decreased from 2000 to 2017 (272 per live birth to 177 per 100,000 live births) but has not yet reached the SDGs target globally [4]. Compared with the MMR trend from 2000 to 2017 in the Southeast Asia region, Indonesia has the highest Maternal Mortality Rate. The lowest is in Singapore (8 per 100,000 live births). Moreover, at the global level, there are at least 830 maternal deaths in the world every day [4].

Previous studies found that 14.3% of maternal mortality was due to abortion bleeding and uterine rupture, followed by eclampsia in 12.5% [5, 6]. On the other hand, other causes of maternal death were infection (sepsis) by 10.7%, then abortion and embolism by 7.9%. The rest are of unknown reasons [7]. We handled incorrect and on-time maternal deaths caused by obstetric complications. About 15% of pregnancies/deliveries have difficulties; they have required quality service readiness at all times. Quick access to quality emergency services is needed, as some complications require emergency care within hours, especially in hospitals, to get complete delivery services [5, 6]. Delay in assisting can cause maternal death. A previous study found at least three delays to cause maternal death: late in making decisions, late arrival at the referral place, and late getting help at the referral place [8].

Several lessons inform caused delays in delivery assistance in eastern Indonesia by access to health facilities and costs incurred during childbirth [9, 10]. Moreover, in the territory of Indonesia, the distribution of hospitals in each region is imbalanced. The disparity in hospital availability also affects the accessibility to delivery at the hospital [11, 12]. Besides, the cost is quite expensive to influence mothers to take advantage of hospital services, especially in Eastern Indonesia [10, 13].

One of the Indonesian government’s policies is to achieve universal health coverage to overcome these conditions. The government does it through the National Health Insurance (NHI). NHI is a form of financial guarantee when the mother wants to give birth in the hospital. The NHI contributes by making it easier for them to access finance, reducing their labor burden [14–16]. Several studies have reported that the NHI can increase women’s access to childbirth in the hospital [15, 17, 18]. This situation is the basis for knowing the importance of differences in health insurance ownership among women who give birth in eastern Indonesia hospitals. This study’s results are significant for policymakers to encourage policies that focus on increasing deliveries to hospitals. This study aimed to analyze health insurance ownership disparities in hospital delivery in Eastern Indonesia based on the background description.

## Materials and methods

### Data source

The author conduct study using secondary data from Indonesian Demographic and Health Survey (IDHS) in 2017. The IDHS was part of the international Demographic and Health Survey (DHS) program conducted by the Inner City Fund (ICF). The IDHS was a crossectional survey. The study population was women aged 15–49 years old who had given birth in the last five years in Eastern Indonesia. The study covered all provinces in Eastern Indonesia, namely East Nusa Tenggara, Maluku, North Maluku, West Papua, and Papua [19]. The study takes samples through stratification and multistage random sampling methods. By the procedure, the study employs a weighted sample size of 2299 respondents.

### Outcome variable

The study defines hospital-based birth delivery as a delivery carried out in hospitals, government, and private hospitals. Hospital-based birth consists of two categories: no and yes.

### Exposure variables

Health insurance ownership was the respondent’s recognition of owned insurance, whether run by the government or the private sector. Health insurance ownership consists of two categories, namely uninsured and insured.

### Control variables

Apart from health insurance ownership, other variables analyzed as control variables were province, residence type, age group, marital status, education level, employment status, parity, and wealth status. The study selected control variables based on previous studies on the same theme [20–22].

The provinces consist of East Nusa Tenggara, Maluku, North Maluku, West Papua, and Papua. Meanwhile, the residence type consists of two categories, namely urban and rural. This categorization refers to Statistics Indonesia. The age group consists of seven classes in 5 years, namely 15–19, 20–24, 25–29, 30–34, 35–39, 40–44, and 45–49. Marital status consists of three categories: never in a union, married/living with a partner, and widowed/divorced. Education level was the respondent’s recognition of the last diploma they have. Meanwhile, education level consists of four categories: no education, primary, secondary, and higher. Parity was the number of living children that have been born. Parity consists of three types, namely primiparous (≤ 1), multiparous [2–4], and grand multiparous (> 4) [23].

The IDHS calculated wealth status based on the wealth quintile owned by a household. Households were scored based on the numbers and types of items they had, from televisions to bicycles or cars, and housing characteristics, such as drinking water sources, toilet facilities, and primary building materials for the house’s floor. The IDHS calculated the score using principal component analysis. National wealth quintiles were arranged based on household scores for each person in the household and divided by the distribution into the same five categories, accounting for 20% of the population. Wealth status consists of five classes: the poorest, poorer, middle, richer, and the richest [24].

### Statistical analysis

The initial stage analysis used chi-square to analyze the disparity characteristics of health insurance ownership and other variables. In the final stage, because of the dependent variable’s nature, binary logistic regression was used to determine health insurance ownership disparities and determine the adjusted odds ratio (AOR) with a 95% confidence interval (CI). The binary logistic regression used “hospital delivery = no” as references. The authors carry out all stages of statistical analysis using SPSS 21.

### Ethical approval

The 2017 IDHS has obtained ethical clearance from the National Ethics Committee in Indonesia. The authors deleted all the respondents’ identities from the dataset. Respondents have provided written approval for their involvement in the study. The researcher has obtained permission to use the 2017 IDHS data through the website: https://dhsprogram.com for this study.

## Results

The analysis results found that, on average, women in Eastern Indonesia in 2017 made hospital deliveries of 28.5%. Meanwhile, the average health insurance ownership in Eastern Indonesia in 2017 was 61.5%.

### Descriptive analysis

Table 1 displays descriptive statistics of health insurance ownership of respondents in Eastern Indonesia. Based on a hospital delivery, it shows that uninsured women rule both categories of hospital delivery. Based on the province, uninsured women occupy all regions. Meanwhile, based on the type of place of residence, uninsured women also dominate in both areas, with a more significant proportion of uninsured in rural areas.

**Table 1:** Descriptive statistics of health insurance ownership of respondents in Eastern Indonesia in 2017 (*n* = 2299)

Variables	Health Insurance Ownership				
	Uninsured		Insured		***p***-value
	n	%	n	%	
Hospital delivery					< 0.001
No	707	79.8%	179	20.2%	
Yes	936	66.2%	477	33.8%	
Province					< 0.001
East Nusa Tenggara	601	71.6%	238	28.4%	
Maluku	480	72.9%	178	27.1%	
North Maluku	292	79.1%	77	20.9%	
West Papua	111	57.5%	82	42.5%	
Papua	159	66.3%	81	33.8%	
Type of place of residence					< 0.001
Urban	332	51.6%	312	48.4%	
Rural	1311	79.2%	344	20.8%	
Age group					< 0.001
15–19	62	71.3%	25	28.7%	
20–24	285	77.9%	81	22.1%	
25–29	409	74.2%	142	25.8%	
30–34	418	71.3%	168	28.7%	
35–39	296	67.1%	145	32.9%	
40–44	128	62.4%	77	37.6%	
45–49	45	71.4%	18	28.6%	
Marital status					< 0.001
Never in union	20	74.1%	7	25.9%	
Married/Living with partner	1553	71.2%	629	28.8%	
Widowed/Divorced	70	77.8%	20	22.2%	
Education Level					< 0.001
No education	72	87.8%	10	12.2%	
Primary	526	82.4%	112	17.6%	
Secondary	836	72.3%	320	27.7%	
Higher	209	49.4%	214	50.6%	
Employment status					< 0.001
Unemployed	816	73.0%	302	27.0%	
Employed	827	70.0%	354	30.0%	
Parity					< 0.001
Primiparous	391	64.1%	219	35.9%	
Multiparous	943	73.3%	343	26.7%	
Grandmultiparous	309	76.7%	94	23.3%	
Wealth status					< 0.001
Poorest	1204	81.9%	266	18.1%	
Poorer	237	62.7%	141	37.3%	
Middle	95	47.0%	107	53.0%	
Richer	80	48.8%	84	51.2%	
Richest	27	31.8%	58	68.2%	

According to the age group, uninsured women led in all age groups categories. Based on marital status, uninsured women ruled all types. Moreover, uninsured women occupied all classes based on education level, except for the higher education group, which insured women dominated.

Table 1 shows that the uninsured women prevalence in all employment status categories. Meanwhile, based on parity, uninsured women rule in all parity types. Finally, based on wealth status, the uninsured women ruled in the poorest and poorer categories; on the other hand, the insured women led in the middle, richer, and the richest categories.

### Multivariable analysis

Table 2 shows that insured women are 1.426 times more likely than uninsured women to have hospital delivery (AOR 1.426; 95% CI 1.426–1.427). This analysis indicates that having health insurance will be more likely to have birth delivery in the hospital in eastern Indonesia for hospital delivery. There is still a disparity between insured and uninsured women in hospital-based birth deliveries in the east of Indonesia. Insured women are nearly one and a half times more likely than uninsured women to give birth in a hospital.

**Table 2:** The results of binary logistic regression of hospital delivery in Eastern Indonesia in 2017 (*n* = 2299)

Variables	Hospital Delivery			
	***p***-value	AOR	95% CI	
			Lower Bound	Upper Bound
Health insurance: No	–	–	–	–
Health insurance: Yes	< 0.001	1.426	1.426	1.427
Province: East Nusa Tenggara	–	–	–	–
Province: Maluku	< 0.001	0.477	0.476	0.477
Province: North Maluku	< 0.001	0.443	0.443	0.443
Province: West Papua	< 0.001	1.068	1.067	1.068
Province: Papua	< 0.001	1.208	1.207	1.208
Type of place: Urban	–	–	–	–
Type of place: Rural	< 0.001	0.376	0.375	0.376
Age group: 15–19	–	–	–	–
Age group: 20–24	< 0.001	0.581	0.580	0.581
Age group: 25–29	< 0.001	0.640	0.640	0.641
Age group: 30–34	< 0.001	0.934	0.933	0.935
Age group: 35–39	< 0.001	1.404	1.403	1.406
Age group: 40–44	< 0.001	2.555	2.552	2.558
Age group: 45–49	< 0.001	2.356	2.352	2.359
Marital: Never in union	–	–	–	–
Marital: Married/Living with partner	< 0.001	1.342	1.340	1.344
Marital: Widowed/Divorced	< 0.001	0.690	0.688	0.691
Education: No education	–	–	–	–
Education: Primary	< 0.001	3.379	3.375	3.383
Education: Secondary	< 0.001	5.175	5.169	5.182
Education: Higher	< 0.001	10.225	10.212	10.238
Employment: Unemployed	–	–	–	–
Employment: Employed	< 0.001	0.954	0.954	0.955
Parity: Primiparous	–	–	–	–
Parity: Multiparous	< 0.001	0.610	0.610	0.610
Parity: Grandmultiparous	< 0.001	0.462	0.462	0.462
Wealth: Poorest	–	–	–	–
Wealth: Poorer	< 0.001	1.642	1.641	1.642
Wealth: Middle	< 0.001	2.208	2.207	2.210
Wealth: Richer	< 0.001	1.944	1.942	1.945
Wealth: Richest	< 0.001	1.626	1.624	1.627

Note: 95% CI; *AOR* adjusted odds ratio

The adjusted odds ratios for the health insurance ownership variable are smaller than the unadjusted odds ratios. The value of unadjusted odds ratios is 2.013 (OR 2.013; 95% CI 1.652–2.453).

Apart from health insurance ownership, all other independent variables analyzed were significant determinants of hospital delivery in Eastern Indonesia. According to the province, women in Maluku and North Maluku are less likely than women in East Nusa Tenggara to carry out hospital delivery. Meanwhile, West Papua and Papua women have a higher probability than women in East Nusa Tenggara for hospital delivery.

Based on the age group, women in 20–24, 25–29, and 30–34 age groups were less likely than women in the 15–19 age group to have hospital delivery. Meanwhile, women in the 35–39, 40–44, and 45–49 age groups had a higher chance than women in the 15–19 age group to have hospital delivery.

Married/living with partner women were 1.342 times more likely than never in union women to undertake hospital delivery (AOR 1.342; 95% CI 1.340–1.344). Moreover, widowed/divorced women were 0.690 times less likely than never in union women to do hospital delivery (AOR 0.690; 95% CI 0.688–0.691).

Women with primary education are 3.379 times more likely than no educated women to have hospital delivery (AOR 3.379; 95% CI 3.375–3.383). Women with secondary education have a 5.175 times chance of education for hospital delivery (AOR 5.175; 95% CI 5.169–5.182). Finally, women with higher education are 10.225 times more likely than no education women to undertake hospital delivery (AOR 10.225; 95% CI 10.212–10.238). This analysis shows that the better the education level, the more likely it is for women in Eastern Indonesia to make hospital delivery.

Employed women were 0.954 times less likely than unemployed women for hospital delivery (AOR 0.954; 95% CI 0.954–0,955). This information shows that unemployment is a protective factor for women in Eastern Indonesia to do hospital delivery.

According to parity, multiparous women are 0.610 times more likely than women who do not have children or have one child to undertake hospital delivery (AOR 0.610; 95% CI 0.610–0.610). Meanwhile, grand multiparous women were 0.462 times less likely than women who do not have children or have one child to undertake hospital delivery (AOR 0.462; 95% CI 0.462–0.462). These multivariate test results inform that the more children who have been born, the less likely it is for women in Eastern Indonesia to have hospital delivery.

Table 2 informs that according to wealth status, women in a more deficient category were 1.642 times more likely than the poorest women to do hospital delivery (AOR 1.642; 95% CI 1.641–1.642). Women in the middle category were 2.208 times more likely than the poorest women for hospital delivery (AOR 2.208; 95% CI 2.207–2.210). The richer category women were 1.944 times more likely than the poorest women to do hospital delivery (AOR 1.944; 95% CI 1.942–1.945). Finally, women in the wealthiest category are 1.626 times more likely than the poorest women to do hospital delivery (AOR 1.626; 95% CI 1.624–1.627). This information shows that all wealth status categories have a higher probability of making hospital delivery than the poorest.

## Discussion

The analysis found that having health insurance will be more likely to have birth delivery in the hospital in Eastern Indonesia to carry out hospital delivery. This analysis results in line with the objectives of Indonesia’s health financing policies that seek to realize National Health Insurance (NHI) with total coverage to minimize the barrier to health financing in Indonesia [15, 25]. This study’s results align with several previous studies on health insurance in various countries [26–28].

The results of this study provide a clear policy direction. The Indonesian government can push for policies that focus on increasing public participation in National Health Insurance to increase childbirth in eastern Indonesia. The Indonesian government can also optimize local governments’ role, for example, by conducting cost-sharing assistance for National Health Insurance contributions for poor people [25].

For eastern Indonesia, having health insurance is not enough because it will only cover service costs. Meanwhile, east Indonesia has extreme geographical characteristics, including archipelagic topography, making transportation costs expensive [10, 11, 29]. These characteristics often make health development in this region lag behind other Indonesian areas [30, 31]. Other policy interventions are still needed to complement the National Health Insurance policy.

In addition to health insurance ownership as exposure, this study also finds control variables as factors that also affect hospital delivery in eastern Indonesia. The study results inform that there are still disparities in hospital utilization between regions (provinces) for delivery. The information on this study’s results is consistent with previous studies that inform that this disparity starts from an unbalanced input of health resources [32, 33]. On the other hand, the Papua Health Insurance, initiated by the local governments in West Papua and Papua, has proven to encourage hospital delivery [10]. West Papua and Papua Provinces have better utilization of hospital delivery services than other provinces in Eastern Indonesia.

The research analysis found that the age group is one of the determinants of women in Eastern Indonesia for hospital delivery. The older a woman is, the higher the chances of having her delivered to the hospital. Perhaps this is related to self-confidence because of previous experience or because pregnant women are increasingly aware of pregnancy and childbirth as they age [34]. Other authors also found age as a factor related to hospital delivery. The authors conduct the study in 34 countries in sub-Saharan Africa [35].

The multivariate analysis results found that having a partner (married/living with a partner) will be more likely for women in Eastern Indonesia to undertake hospital delivery. Psychologically, having a partner makes a woman have a place to share the burden, including the burden of financing related to childbirth costs [36]. Moreover, several previous studies have also found husbands’ involvement has increased health services utilization [37, 38]. In the Indonesian context, this condition follows local values and local culture, which views a pregnant woman without a partner as a disgrace [39, 40], so pregnant women without a partner tend to hide their pregnancy and childbirth.

The study found education will be more likely to give birth for women in Eastern Indonesia to undertake hospital delivery. The better the level of education, the higher the possibility of women in Eastern Indonesia launching hospital delivery. The higher the woman’s education, the more independent she is in determining what is best [41]. This finding is in line with the results of previous studies in Bangladesh and Ethiopia [42, 43]. Generally, several lessons that inform better education levels are a strong determinant of better health output [19, 44, 45]. Otherwise, poor education is known as a barrier to achieving better health output [16, 46].

The study found unemployed will be more likely to have a birth for women in Eastern Indonesia to conduct hospital delivery. It is possible that this situation can occur because unemployed women have a better time to prepare for delivery, including better choices of places for delivery. This study’s results align with previous studies in Indonesia that analyzed IDHS data in 2013 [47].

According to parity, the results found that the more children who have been born, the less likely women in Eastern Indonesia are to have hospital delivery. Women in Eastern Indonesia with higher parity are likely to be more confident about delivering at a facility other than a hospital. Previous birth experiences also influence the choice of place of delivery [35]. an earlier study in Southern Ethiopia also confirmed similar findings [43].

According to wealth status, the study found that all wealth status categories have a higher probability than the poorest to make hospital delivery. In other words, poverty is a barrier for women in Eastern Indonesia to undertake hospital delivery. This situation is what the government wants to overcome, removing the public’s financing barrier to access health services by releasing the NHI policy [25]. Several previous studies confirmed wealth status as a strong predictor of better health performance [24, 48–50].

### Study limitation

This study carries out limitations as a consequence of the use of secondary data received. Studies analyze superficial data. The study does not consider cultural factors and beliefs known in previous studies to influence women’s choice to deliver in the hospital or not [51–53]. On the other hand, the 2017 IDHS is a cross-sectional survey, and hence the findings drawn are not causal.

## Conclusions

The authors concluded that health insurance ownership disparities for hospital deliveries in Eastern Indonesia were based on the study results. Insured women have a better chance than uninsured women for hospital delivery.

The implication of this study’s results is related to the health financing policy carried out by the Indonesian government through a social insurance mechanism called National Health Insurance. If the government wants to encourage deliveries to hospitals, the government must formulate policies to increase Eastern Indonesia’s community participation in National Health Insurance.

## Data Availability

The authors cannot share data because a third party and authors who own the data do not have permission to share it. The 2017 IDHS data set name requested from the ICF (‘data set of childbearing age women’) are available from the ICF contact via https://www.dhsprogram.com for researchers who meet the criteria for access to confidential data.
